# Pneumococcal Endocarditis in a 49-Year-Old Male With Concomitant Myocarditis, Septic Spinal Arthritis, and Paraspinal Myositis: A Case Report

**DOI:** 10.7759/cureus.71491

**Published:** 2024-10-14

**Authors:** Timothy Moore, Brandon B Boyle, Andrej Urumov, Scott Calder, Wayne A Martini

**Affiliations:** 1 Emergency Medicine, Mayo Clinic Alix School of Medicine, Scottsdale, USA; 2 Emergency Medicine, Northwestern University Feinberg School of Medicine, Chicago, USA; 3 Emergency Medicine, Mayo Clinic, Phoenix, USA

**Keywords:** aortic valve replacement, bacterial septicemia, emergency medicine, endocarditis, multisystem infection, myocarditis, sepsis, septic emboli, streptococcus pneumoniae

## Abstract

Pneumococcal infective endocarditis (PIE) is a rare but serious infection often presenting with systemic complications such as septic emboli, myocarditis, and septic arthritis. This case report highlights a 49-year-old male who presented with acute-on-chronic lower back pain and fever, later diagnosed with PIE complicated by septic spinal arthritis, paraspinal myositis, and developing myocarditis.

A 49-year-old male presented to the emergency department (ED) with worsening back pain and fever after treatment failure for suspected pyelonephritis. Laboratory studies revealed leukocytosis, hypercalcemia, and acute kidney injury, while magnetic resonance imaging (MRI) identified septic facet arthritis and abscess formation. A transesophageal echocardiogram (TEE) revealed aortic valve vegetations consistent with infective endocarditis (IE). The patient required aortic valve replacement and prolonged hospital stay due to sepsis and respiratory failure. He recovered within 12 weeks with only moderate residual heart failure symptoms.

This case highlights the medical complexities and difficulties of treating IE, as well as the critical importance of having it on a wide differential due to increased morbidity and mortality with delay of diagnosis, antibiotics, and surgical intervention.

## Introduction

Infective endocarditis (IE) is a severe condition often complicated by systemic manifestations that can lead to a range of clinical sequelae, such as septic emboli resulting in myocarditis, septic arthritis, and myositis. *Streptococcus pneumoniae* is a rare cause in contemporary cases of endocarditis, accounting for <3% of cases since the wider use of penicillin in the 1940s [1]. Infective endocarditis due to *Streptococcus* spp. has declined, and *Staphylococcus aureus* has become the first leading cause of this disease, representing more than 25% of cases [2]. Despite its rarity, *Streptococcus pneumoniae* continues to pose significant risks, particularly in middle-aged men with a history of liver disease, alcohol abuse, intravenous (IV) drug use, and/or valve replacement, where it most commonly affects the aortic valve and/or the mitral valve [3-5].

*Streptococcus pneumoniae* infective endocarditis, also known as pneumococcal infective endocarditis (PIE), is notorious for its fulminant course and poor prognosis, requiring early surgical intervention [3]. The reported mortality rate for PIE is around 21%, and the associated complications, such as septic emboli, meningitis, and heart failure, further complicate clinical management and prognosis ​[3,5]. This report aims to contribute to the existing publications by providing a literature review along with the clinical course, management, and outcomes of a patient with a rare and severe presentation of IE. This case report presents a rare and complex instance of *Streptococcus pneumoniae* infective endocarditis in a 49-year-old male with concomitant myocarditis, septic spinal arthritis, and paraspinal myositis.

## Case presentation

We introduce a 49-year-old male with a past medical history of hypertension and alcohol abuse who presented to the emergency department (ED) with worsening back pain, fever, chills, and night sweats, with bilateral flank and lumbothoracic back pain. The symptoms deteriorated since onset eight days prior to ED arrival. He described the pain as sharp, severe, and radiating to his suprapubic region and testicles. His vital signs were significant for a heart rate of 110 beats/minute at triage, but otherwise, he was afebrile and normotensive, with a respiratory rate of 15 breaths/minute and oxygen saturation of 96% on room air.

He had previously been seen at an outside hospital ED two days prior where an outside computed tomography (CT) of the abdomen and pelvis with intravenous (IV) contrast, complete blood count (CBC), complete metabolic panel (CMP), urinalysis, and urine culture were reviewed. The CT failed to reveal acute findings, CBC showed no anemia or leukocytosis, and kidney function was normal. His urinalysis showed no evidence of infection.

The patient was discharged with a presumptive diagnosis of acute pyelonephritis and treated with a course of cephalexin. Upon this secondary presentation, on general examination, the patient appeared to be in moderate distress with diaphoresis. On initial evaluation, the patient was sitting on the edge of the bed due to back discomfort. His physical examination showed reproducible spinal and paraspinal tenderness along the T8-L4 vertebra and no abdominal tenderness. Cardiac examination showed tachycardia with a regular rhythm and a new decrescendo early-diastolic blowing murmur, best heard on the left lower sternal border, around the third and fourth intercostal spaces. Lungs were clear to auscultation bilaterally.

Due to the patient's increased pain and return visit, a broader differential was considered in this second presentation to the ED. A septic workup including CBC, CMP, blood cultures, lactate, viral panel, portable chest X-ray, C-reactive protein (CRP), urinalysis, and urine cultures were ordered for the patient. He was treated for sepsis empirically with cefepime and vancomycin. The patient's abnormal laboratory results are reported in Table 1.

**Table 1:** Abnormal emergency department laboratory parameters BUN: blood urea nitrogen, CRP: C-reactive protein

Laboratory parameter	Patient value	Normal reference range
White blood cell count	17.4 × 10⁹/L	3.4-9.6 × 10⁹/L
Hemoglobin	12.7 g/dL	13.2-16.6 g/dL
Serum creatinine	1.35 mg/dL	0.6-1.2 mg/dL
BUN	29 mg/dL	8-24 mg/dL
CRP	266 mg/L	≤8 mg/L

**Figure 1:**
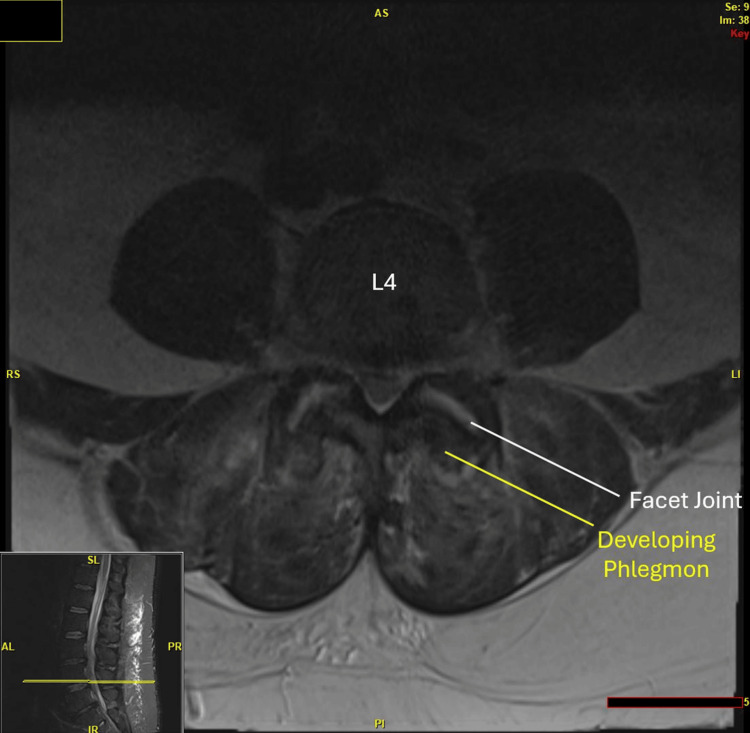
This region likely corresponds to areas of inflammation or developing phlegmon or abscess adjacent to the L4-L5 facet joints. The collection seen along the posterior aspect of the left L4-L5 facet joint likely communicates with the joint space itself, indicating that the abscess or phlegmon is either involving or affecting the joint capsule.

**Figure 2:**
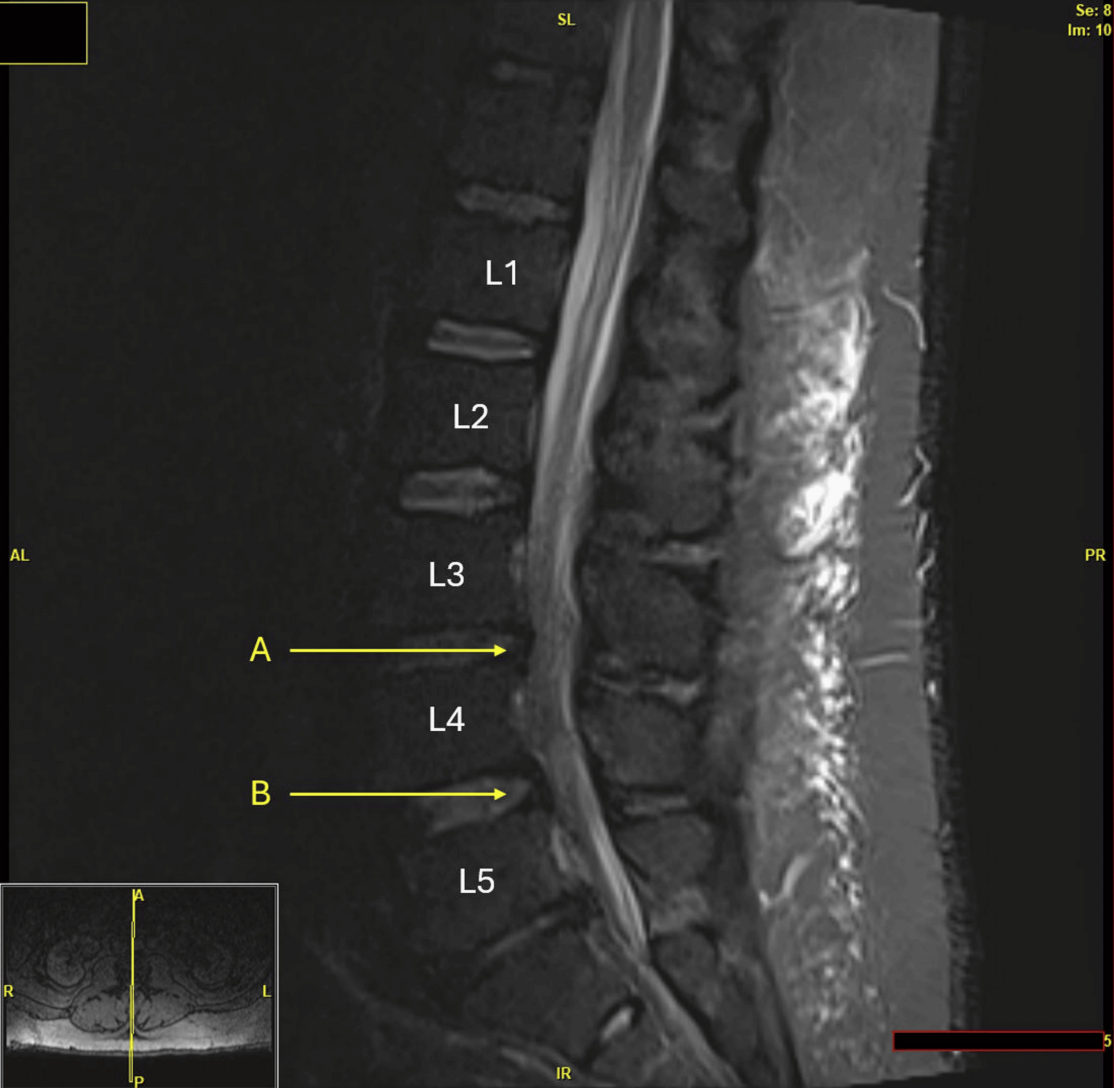
At the L3-L4 level (arrow A), there is mild spinal canal stenosis and mild bilateral foraminal narrowing, mainly caused by a broad-based disc bulge, with potential contribution from acute epidural inflammatory changes. At the L4-L5 level (arrow B), there is moderate spinal canal stenosis and mild bilateral foraminal narrowing, resulting from a combination of chronic broad-based disc bulge, facet hypertrophy, and acute epidural inflammatory changes. No definitive evidence of an epidural abscess is observed.

New clinical findings and his worsening back pain warranted a repeat CT of the abdomen and pelvis, which was significant for hepatomegaly. A scrotal ultrasound with Doppler was performed, which showed no signs of epididymitis or testicular torsion. A magnetic resonance imaging (MRI) of the lumbar spine was ordered, and the patient was admitted for management of sepsis of unclear origin. The lumbar spine MRI demonstrated extensive posterior paraspinal musculature phlegmonous changes (Figure 1), micro-abscesses, and central canal stenosis (Figure 2), without drainable abscess or osteomyelitis. A transthoracic echocardiogram was performed for this new murmur, which showed severe eccentric aortic regurgitation with possible perforation of the right coronary cusp. Cardiology was consulted, and a transesophageal echocardiogram (TEE) was performed, which showed small mobile echo-densities on the aortic valve with torn right coronary cusp (Video 1) and severe aortic regurgitation (Video 2) with an ejection fraction of 60%.

**Video 1:** Transesophageal echocardiogram revealing small mobile echo-densities on the aortic valve with torn right coronary cusp

**Video 2:** Transesophageal echocardiogram revealing the aortic valve with torn right coronary cusp and severe aortic regurgitation

The blood cultures grew *Streptococcus pneumoniae*, which confirmed the suspected acute bacterial endocarditis causing septic emboli. Antibiotic treatment was continued. On hospital day 6, he underwent aortic valve replacement surgery. The patient's hospital course was complicated by kidney failure requiring dialysis as well as vasopressor and ventilatory support. Despite his prolonged hospital stay, at follow-up evaluation at four weeks, he made significant improvement in activities of daily living and was no longer requiring dialysis. At 12 weeks, he was experiencing a near full recovery, although he still had significant symptoms of heart failure.

## Discussion

*Streptococcus pneumoniae* is a gram-positive bacterium that commonly causes community-acquired pneumonia. Infection usually begins with colonization of the oropharynx and nasopharynx, spreading to the lower airways [6]. Pneumonia typically develops when predisposing risk factors such as smoking, alcohol use, IV drug use, or increased age are present. For a small subset of these individuals (about 0.3%), the bacteria may disseminate into the bloodstream, spreading to the heart and forming vegetations [4]. These vegetations can then cause septic emboli to the lungs (if right-sided vegetation) or to other organ systems (if left-sided vegetation), leading to a variety of complications, including meningitis and septic arthritis.

Unlike other forms of IE, PIE often lacks classic signs such as Osler nodes, Janeway lesions, or Roth spots [1]. Instead, it frequently begins with signs of systemic infection, such as fever, malaise, and chills, which may progress to pneumonia and/or meningitis, often resulting in initial misdiagnoses of pneumonia, sepsis, or COVID-19. The thromboembolic nature of the vegetations in PIE can also lead to septic arthritis, osteomyelitis, endophthalmitis, liver and spleen abscesses, and non-resolving otitis media [5].

The diagnostic triad specific to PIE, known as "Osler's triad," includes pneumonia, meningitis, and endocarditis and is seen in approximately 26.1% of cases [5,7]. Unfortunately, this triad is often missed because cardiac symptoms tend to develop late in the disease course, leading physicians to consider non-cardiac causes [1]. While PIE most commonly affects the aortic valve (53.2% of cases) and mitral valve (40.5% of cases), right-sided valvular infections can also occur and may present without classic signs such as cardiac murmurs, petechiae, or splinter hemorrhages [5,8,9]. In such patients, there may be a "tricuspid syndrome," characterized by recurrent pulmonary events, microscopic hematuria, and anemia, although the prevalence of this syndrome is not well-documented [7].

The obscured clinical picture of PIE can lead to significant diagnostic delays. Although the average delay in diagnosis is not well-documented, the median diagnostic delay for PIE is approximately 14 days from hospital admission [10-12]. Delayed treatment of PIE is particularly dangerous because of the disease's rapid progression. PIE is more likely to progress to shock and heart failure compared to other forms of IE, with a median time from symptom onset to heart surgery of about 14 days, compared to 69 days in non-pneumococcal endocarditis [13]. Similarly, patients with PIE have an increased five-year mortality rate of 39.3%, which rises to nearly 60% when Osler's triad is present [14]. Both rates are considerably higher than the 17.9% mortality rate observed in non-pneumococcal endocarditis [13].

The lack of initial recognition of IE in the disease course often leads to delays in appropriate medical therapy. A study comparing early versus late (before versus after four days) echocardiograms in IE patients found that those with late echocardiograms had a non-significant increase in severe heart valve destruction (P = 0.336) but a significant increase in the need for post-IE valve surgery (P = 0.04) [15].

Once suspected, targeted antibiotic therapy must begin immediately as rapid intervention will help prevent the pathogen's invasion and embolic dissemination. For penicillin-susceptible strains, the American Heart Association (AHA) recommends a four-week course of penicillin, ampicillin, or ceftriaxone, often combined with gentamicin for the first two weeks [16,17]. Unfortunately, resistance to beta-lactams, particularly penicillin, is rising, with resistance rates varying by infection type and location [18]. For pneumococcal strains affecting the central nervous system (CNS), resistance can reach up to 85.7%, which is especially concerning given that 40.5% of patients with PIE also experience CNS involvement [5]. In such cases, the AHA recommends high-dose ceftriaxone, with vancomycin as an additional option [17,18]. Fortunately, effective antibiotic therapy can significantly reduce the risk of IE-induced stroke by 50% within just one week [19].

Due to the rapid and aggressive nature of PIE, surgical intervention to remove vegetations and/or replace damaged heart valves should be strongly considered. The American College of Cardiology (ACC) and AHA recommend surgery for IE patients presenting with heart failure, uncontrolled infection, heart block, or persistent vegetations [20]. Surgical intervention in patients with heart failure significantly improves outcomes, with in-hospital mortality of 21% compared to 45% for those treated medically [20]. Valvular replacement, particularly of the aortic valve, is common, with 50%-64.3% of patients requiring this surgery [5]. The mean time from symptom onset to surgery is about 14 days for PIE, significantly shorter than the 69 days observed in non-pneumococcal endocarditis [13].

## Conclusions

Pneumococcal infective endocarditis is a rapidly progressing disease with high morbidity and mortality rates, often presenting atypically and lacking classic bacterial endocarditis symptoms. This can lead to diagnostic delays, allowing the bacteria to spread to challenging areas such as the CNS. Therefore, clinicians must maintain a high index of suspicion for PIE in patients presenting with systemic signs such as fever and chills, especially in the presence of a new heart murmur and predisposing factors such as a history of alcohol or intravenous drug addiction. Early echocardiography is crucial for patients with systemic infection signs and a relevant history, enabling early detection of vegetations that, if treated promptly, can prevent heart failure and septic embolization.

Clinicians should recognize that antibiotics alone may not suffice for treating PIE. Due to the rapid spread and frequent diagnostic delays, heart valve damage is of significant concern. Prompt intervention with appropriate antibiotics and, if necessary, early surgical intervention can markedly improve patient outcomes and prevent serious complications.
